# State-dependent and cell type-specific temporal processing in auditory thalamocortical circuit

**DOI:** 10.1038/srep18873

**Published:** 2016-01-05

**Authors:** Shuzo Sakata

**Affiliations:** 1 Strathclyde Institute of Pharmacy and Biomedical Sciences, University of Strathclyde, 161 Cathedral Street, Glasgow G4 0RE, UK

## Abstract

Ongoing spontaneous activity in cortical circuits defines cortical states, but it still remains unclear how cortical states shape sensory processing across cortical laminae and what type of response properties emerge in the cortex. Recording neural activity from the auditory cortex (AC) and medial geniculate body (MGB) simultaneously with electrical stimulations of the basal forebrain (BF) in urethane-anesthetized rats, we investigated state-dependent spontaneous and auditory-evoked activities in the auditory thalamocortical circuit. BF stimulation induced a short-lasting desynchronized state, with sparser firing and increased power at gamma frequency in superficial layers. In this desynchronized state, the reduction in onset response variability in both AC and MGB was accompanied by cell type-specific firing, with decreased responses of cortical broad spiking cells, but increased responses of cortical narrow spiking cells. This onset response was followed by distinct temporal evolution in AC, with quicker rebound firing in infragranular layers. This temporal profile was associated with improved processing of temporally structured stimuli across AC layers to varying degrees, but not in MGB. Thus, the reduction in response variability during the desynchronized state can be seen subcortically whereas the improvement of temporal tuning emerges across AC layers, emphasizing the importance of state-dependent intracortical processing in hearing.

Neocortical circuits show coordinated activity even in the absence of sensory inputs and this coordinated spontaneous activity defines cortical states^1^,^2^,^3^. In one extreme, a “synchronized” state during slow-wave sleep is characterized by slow fluctuations between synchronous and silent population activity, respectively called UP and DOWN states/phases^4^. On the other hand, a “desynchronized” state during attentive state and rapid eye movement sleep is characterized by tonic desynchronized activity^5^.

These cortical states can affect the sensory response properties in many facets^2^,^6^,^7^,^8^,^9^,^10^,^11^,^12^. This includes response amplitudes^6^,^11^,^13^, the size of receptive fields^14^,^15^,^16^,^17^, and noise correlations between pairs of neurons^18^,^19^. As cortical neurons are highly heterogeneous across cortical layers^20^,^21^, the effects of cortical states are also heterogeneous depending on cell classes: with respect to spontaneous activity, different cortical states can be characterized by diverse spontaneous firing across cortical cell types and layers in the barrel^22^,^23^, auditory^24^,^25^ and visual cortices^8^,^13^,^26^. While patterns of sensory evoked activities differ across cortical layers regardless of cortical states^23^,^27^,^28^,^29^, our understanding of state-dependent and cell type-specific sensory processing is still incomplete.

In the auditory system, although previous studies have shown that auditory evoked responses in AC are dependent upon brain state^9^,^16^,^24^,^30^,^31^,^32^,^33^, little is known about cell type or laminar specificity of state-dependent temporal tuning in AC despite the fact that temporal processing is critical for speech recognition^34^ and that impairments in temporal processing have been associated with dyslexia^35^ and aging^36^. Moreover, it still remains elusive as to what extent state dependence of auditory responses in AC is inherited from subcortical areas, such as the medial geniculate body (MGB).

Here we adopted *in vivo* large-scale extracellular recording from both AC and MGB simultaneously with electrical stimulations of the basal forebrain (BF) in urethane-anesthetized rats. Electrical stimulation of BF has been applied in studies of the visual cortex^8^ and offers an excellent experimental model to systematically assess state-dependent neural responses. Although BF stimulation has been applied to induce cortical plasticity in AC over the past decades^37^,^38^, only a few studies^39^ have investigated state-dependent auditory processing with this paradigm. In addition, to the best of our knowledge, no previous study assessed cell type-specific firing across cortical layers using this paradigm. In the present study, we report that AC and MGB are similar in terms of the reduction in spike count variability at stimulus onset during the desynchronized state while cortical narrow spiking cells show increased spike rate. Importantly, AC shows distinct response dynamics, with quicker rebound firing during the desynchronized state. This temporal profile was further associated with improved processing of temporally structured stimuli across AC layers to varying degrees, but not in MGB.

## Results

**Figure 1:**
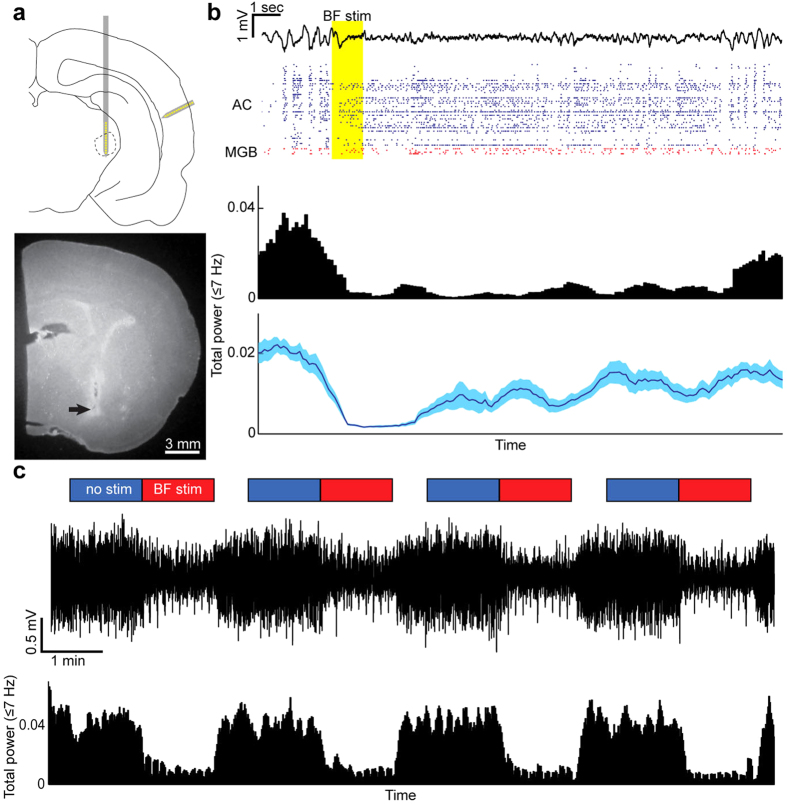
Simultaneous recording from the auditory cortex (AC) and medial geniculate body (MGB) with electrical stimulation of the basal forebrain (BF). (**a**) A schematics of silicon probe insertions (*top*) and a histological image of a stimulation electrode track (*bottom*). (**b**) An example of simultaneous recording from AC and MGB populations with an electrical stimulation of BF. *Top*, Cortical local field potentials (LFPs) and single-unit activities are shown. After BF stimulation (shaded), LFPs displayed fast, small fluctuations for ~10 sec. *Middle*, Total power at ≤7 Hz is shown in this typical example. *Bottom*, An average profile of total power at ≤7 Hz is shown (n = 16). Errors indicate SEM. (**c**) A block design of BF stimulations with auditory stimulus presentations, showing LFPs and total power at ≤7 Hz.

**Figure 2:**
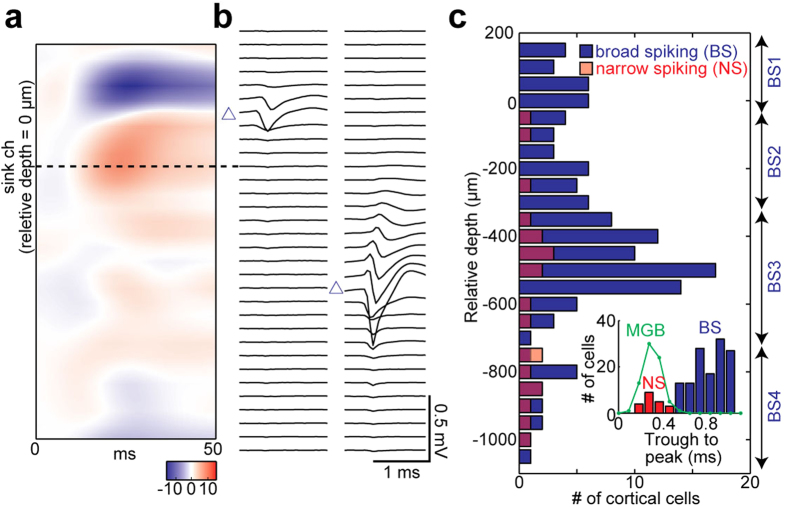
Laminar analysis of simultaneously recorded neurons in AC. (**a**) An example depth profile of current source density. The channel which showed the maximum sink signals was identified as presumptive thalamic recipient layers (L3/4) (0 μm relative depth). (**b**) Examples of mean spike waveforms of simultaneously recorded neurons. Somatic location (triangle) was estimated as the recording site with maximum trough-to-peak amplitude based on mean spike waveforms. (**c**) Distribution of all analyzed single units in AC from 5 rats. Classification of broad spiking (BS) cells based on depths was indicated on the right side. *Inset*, a distribution of trough-to-peak duration. The threshold between broad spiking (BS) and narrow spiking (NS) cells was set at 0.55 ms. Note that MGB cells had a distribution similar to NS cells.

We monitored spontaneous and evoked neural population activity from the auditory cortex (AC) and the medial geniculate body (MGB) simultaneously in urethane-anesthetized rats (n = 5) using linear silicon probes (Fig. 1a). To induce a desynchronized state, the basal forebrain (BF) was electrically stimulated (Fig. 1b). The effect of BF stimulation typically lasted for up to 10 sec. By employing a block design (Fig. 1c), we systematically assessed state-dependent auditory evoked population activity in the auditory thalamocortical circuit. In addition to cortical local field potentials (LFPs) (see below), 151 cortical and 74 thalamic cells were analyzed as single units. To evaluate laminar-dependent effects, cortical cells were further classified into 5 types by assessing spike waveforms and estimating the depth of recorded single units based on spike waveforms and the depth profile of current source density (Fig. 2) (see Methods): narrow spiking (NS) cells (n = 21) and 4 types of broad spiking (BS) cells (BS1, 0 ~ 200 μm, n = 19; BS2, −300 ~ 0 μm, n = 27; BS3, −700 ~ −300 μm, n = 70; BS4, −1100 ~ −700 μm, n = 14). BS1-4 may roughly correspond to layer (L) 2/3, L3/4, L5 and L6, respectively^25^,^40^. Stimulation electrode tracks were histologically localized in the following BF nuclei: the ventral pallidum, the basal part of the substantia innominata, the horizontal limb of the diagonal band, and the magnocellular preoptic nucleus (Fig. 1a and Supplementary Fig. S1).

### Laminar-dependent effects of BF stimulation on cortical local field potentials

**Figure 3:**
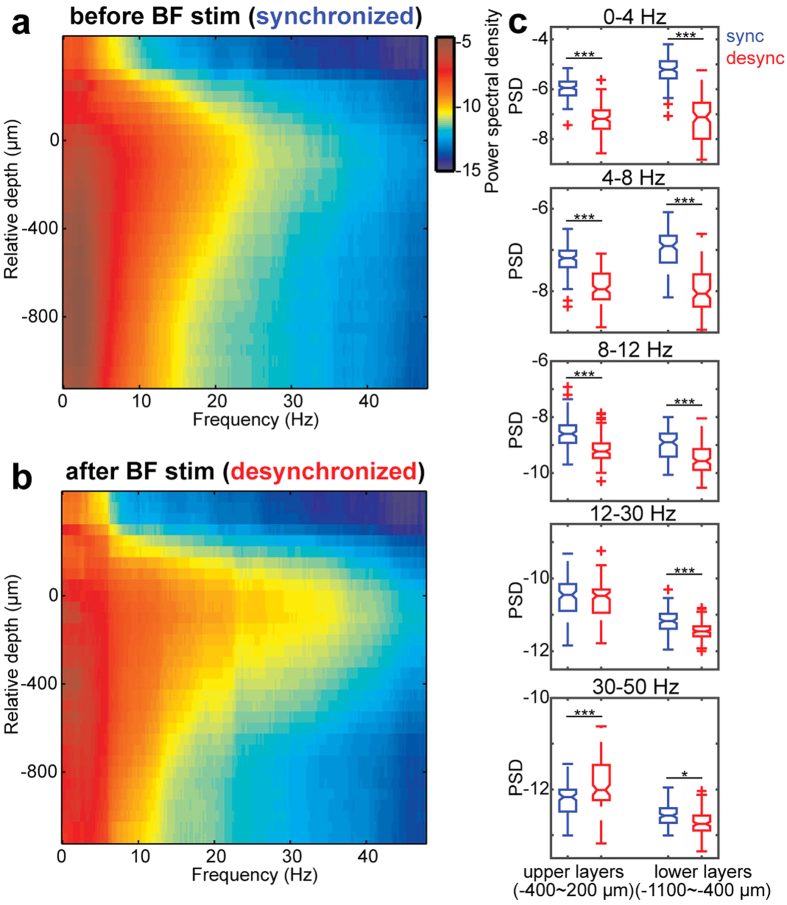
State-dependent and laminar-specific cortical oscillations. (**a,b**) An example depth profile of LFP power spectral density (PSD) before (**a**) and after BF stimulations (n = 21 from a single animal) (**b**). (**c**) Comparisons of PSDs between cortical states (*blue*, desynchronized state; *red*, synchronized state) across frequency bands in different cortical depths from 5 experiments. ***p < 0.0001, *p < 0.05 (two-way ANOVA with HSD test).

To characterize the induced desynchronized state, we began with investigating relationships between cortical states and cortical LFPs across cortical layers (Fig. 3). In Fig. 3a,b, we compared the depth profile of power spectral densities (PSD) in AC before and after BF stimulations (n = 21). Before BF stimulations (Fig. 3a), larger signals at delta frequency (≤4 Hz) were observed particularly in deep channels whereas beta (12–30 Hz) frequency was relatively prominent around presumptive thalamic recipient layers compared to other layers. After BF stimulations (Fig. 3b), the delta component in deep layers decreased whereas the low gamma (30–50 Hz) component increased in presumptive thalamic recipient and supragranular layers (Fig. 3b). This pattern was consistently observed across five experiments (Fig. 3c). In deep layers, effects of BF stimulations were significant across all frequency components assessed (0–4 Hz, F_1,464_ = 783, *p* < 0.0001; 4–8 Hz, F_1,464_ = 416, *p* < 0.0001; 8–12 Hz, F_1,464_ = 158, *p* < 0.0001; 12–30 Hz, F_1,464_ = 25.66, *p* < 0.0001; 30–50 Hz, F_1,464_ = 7.89, *p* < 0.005, two-way ANOVA with post-hoc Tukey’s honest significant different (HSD) test). In superficial layers, low frequency components (up to 12 Hz) were decreased (0–4 Hz, *p* < 0.0001; 4–8 Hz, *p* < 0.0001; 8–12 Hz, *p* < 0.0001) whereas low gamma component was significantly increased by BF stimulations (*p* < 0.0001). These results suggest layer-specific effects of cortical states on network activity in AC.

### Laminar-dependent effects of BF stimulation on thalamocortical spontaneous activity

**Figure 4:**
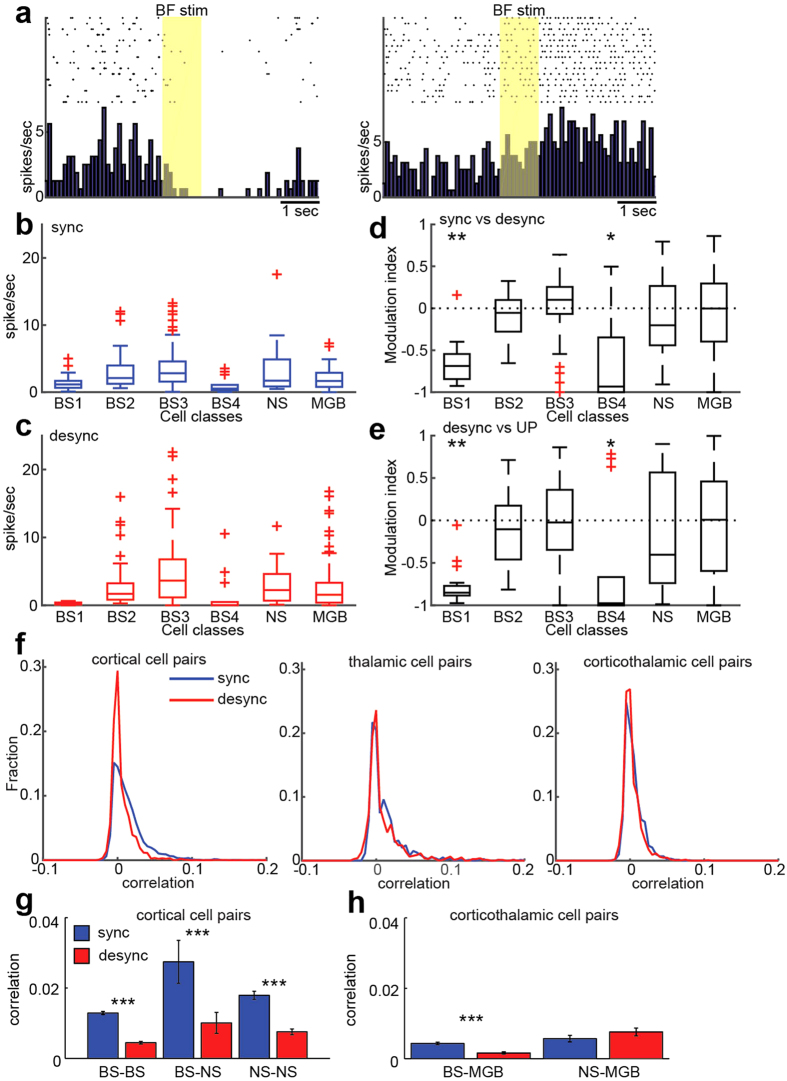
Cell type-specific effects of BF stimulation on spontaneous spiking activity. (**a**) Two examples of modulated cortical neurons (*left*, BS1 cell; *right*, BS3 cell). (**b,c**) Mean firing rate before BF stimulations (synchronized state) (**b**), after BF stimulations (desynchronized state) (**c**). For results of post-hoc multiple comparisons, see Supplementary Tables 1 and 2. (**d**) Modulation index of changes in mean firing rate between the synchronized and desynchronized states. **p < 0.005, *p < 0.05 (signed rank test with Bonferroni correction). (**e**) Modulation index of changes in mean firing rate between the desynchronized state and UP state. The modulation index was computed from (UP – DESYNC)/(UP + DESYNC), where UP and DESYNC were mean firing rate between UP state and the desynchronized state, respectively. **p < 0.005, *p < 0.05 (signed rank test with Bonferroni correction). (**f**) Distribution of spike count correlations between cortical (*left*) (n = 2298, *p* < 0.0001, two-way ANOVA with post-hoc HSD test), thalamic (*middle*) (n = 792, *p* = 0.41), and thalamocortical pairs (*right*) (n = 1316, *p* = 0.25). (**g,h**) Mean spike count correlations between cell classes. ***p < 0.0001 (two-way ANOVA with post-hoc HSD test). Note that asterisks are shown only for comparisons within pairs. For other post-hoc comparisons, see Supplementary Tables 4 and 5.

Next, we characterized state-dependent spontaneous firing in AC and MGB neurons (Fig. 4). Figure 4a shows examples of two cortical neurons whose spontaneous firing was differently modulated by BF stimulation. Typically superficial neurons showed decreased firing in the induced desynchronized state whereas many infragranular neurons exhibited continuous firing, resulting in increased firing, generally consistent with a previous report with electrical stimulation of the pedunculopontine tegmental nucleus under anesthesia and also with results in unanesthetized rats^25^. To quantify this tendency, firstly we compared mean firing rate across cell classes during the synchronized state (i.e., before BF stimulation) (Fig. 4b and Supplementary Table S1). BS1 and BS4 cell groups showed significantly lower firing rate compared to other cortical cell groups (*p* < 0.0005, Kruskal-Wallis test). This trend became stronger during the desynchronized state (Fig. 4c and Supplementary Table S2). Mean firing rate in BS1 and BS4 cell groups was significantly lower than other groups including MGB cells (*p* < 0.0001, Kruskal-Wallis test). Next, we assessed the effect of state changes computing a modulation index (see Methods) (Fig. 4d). BS1 and B4 cell groups significantly decreased their firing rate after BF stimulations (BS1, *p* < 0.005; BS4, *p* < 0.05, signed rank test with Bonferroni correction). Other cell classes showed diverse effects. These results indicate state-dependent and cell type-specific spontaneous firing in the auditory thalamocortical circuit. To further ask whether the desynchronized state can explain as a continuous UP state, we compared firing rate between both conditions (Fig. 4e). Again, BS1 and BS4 cells showed a significant reduction in their firing rate during the desynchronized state (BS1, *p* < 0.005; BS4, *p* < 0.05, signed rank test with Bonferroni correction), suggesting that the desynchronized state differs from UP state of the synchronized state, with respect to suprathreshold spiking activity across cortical layers.

Both states were also characterized by measuring pairwise spike count correlations across recorded neurons (Fig. 4f–h and Supplementary Tables 3–5). The desynchronized state was associated with significantly lower correlations only in cortical cell pairs (F_2,8806_ = 46.2, *p* < 0.0001, two-way ANOVA with HSD test), and not in thalamic pairs (*p* = 0.41) or corticothalamic pairs (*p* = 0.25) (Fig. 4f and Supplementary Table 3). While a significant reduction in correlations can be observed across three different combinations of cortical cells (F_2,4590_ = 3.86, *p* < 0.0001 for all, two-way ANOVA with HSD test) (Fig. 4g and Supplementary Table 4), intriguingly BS-MGB cell pairs showed the reduction in spike count correlations (F_2,2628_ = 13.7, *p* < 0.0001), but not in NS-MGB cell pairs (*p* = 0.32) (Fig. 4h and Supplementary Table 5). Thus, cortical states are characterized by diverse, but cell type-specific population firing.

### Effects of cortical state on onset responses to auditory click stimulus

**Figure 5:**
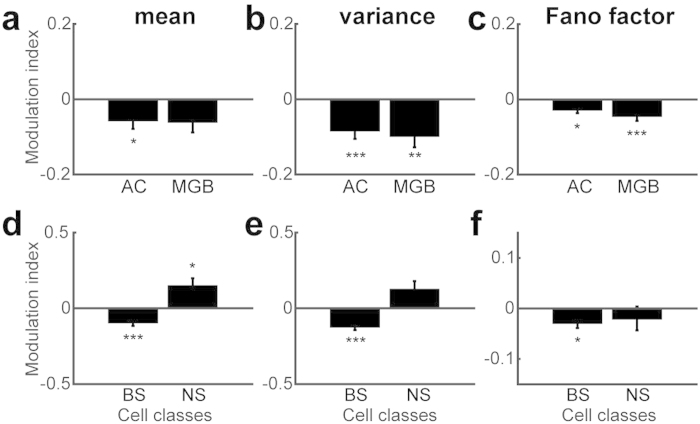
State dependence of trial-by-trial spike count reliability in auditory evoked responses to click stimulus. (**a–c**) Modulation index of mean firing rate (**a**), variance (**b**), and Fano factor (**c**) in AC and MGB. (**d–f**) Modulation index of AC cell classes. *p < 0.05, **p < 0.01, ***p < 0.005 (two-tailed *t*-test with Bonferroni correction).

To investigate how cortical states shape auditory evoked responses to a simple stimulus, single clicks were presented during the synchronized and desynchronized states. 130 cortical and 70 thalamic neurons that showed spiking (0 to 100 ms from onset) in both states were included for the following analysis. We began by analyzing responses in the first 100 ms time window after stimulus onset by measuring mean spike rate, variance, and Fano factor. The change in each measure was quantified by computing a modulation index (see Methods) (Fig. 5), and a significant reduction was observed in all three measures in both AC and MGB during the desynchronized state (*p* < 0.05, two-tailed *t*-test with Bonferroni correction) except mean responses in MGB neurons (*p* = 0.086) (Fig. 5a–c). Intriguingly, mean evoked responses decreased in BS cells (*p* < 0.0005) during the desynchronized state whereas NS cells showed an opposite trend (*p* < 0.05) (Fig. 5d). BS cell population showed significant reduction in variability (*p* < 0.0001) (Fig. 5e) and Fano factor (*p* < 0.05) (Fig. 5f). Thus, with heterogeneous effects on cortical cells, the desynchronized state improved spike count reliability in both AC and MGB.

### State-dependent temporal structure of auditory evoked population activity

**Figure 6:**
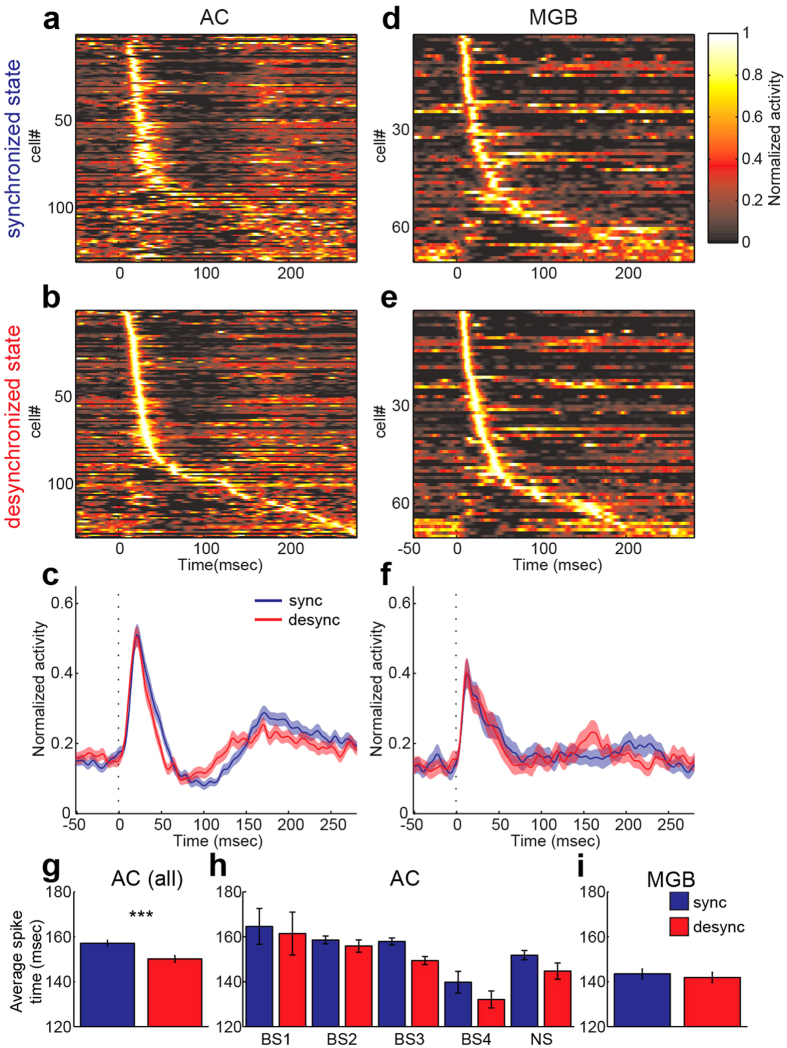
State dependence of response dynamics to click stimulus. (**a–c**) Normalized response profile to click stimulus in the synchronized (**a**) and desynchronized states (**b**) across recorded AC neurons (n = 130). For normalization, mean firing rate was divided by peak firing rate. AC neurons are sorted by peak latency in the desynchronized state. (**c**) Comparison of mean normalized response profiles in AC. *Red*, desynchronized state. *Blue*, synchronized state. Errors indicate SEM. (**d–f**) Normalized response profile to click stimulus in the synchronized (**d**) and desynchronized states (**e**) across recorded MGB neurons (n = 70). MGB neurons are sorted by peak latency in the desynchronized state. (**f**) Comparison of mean normalized response profiles in MGB. *Red*, desynchronized state. *Blue*, synchronized state. Errors indicate SEM. (**g–i**) Mean spike timing in 80–200 ms time window. ***p < 0.0005 (two-tailed *t*-test).

To identify the difference in state-dependent auditory evoked responses between AC and MGB, we looked at the time evolution of evoked population activity in AC (Fig. 6a–c) and MGB (Fig. 6d–f). While neurons in both areas showed diverse response profiles, auditory cortical neurons typically showed a large onset response followed by brief suppression and rebound activation regardless of cortical state (Fig. 6a–c). Notably, this temporal profile in AC appeared slightly quicker during the desynchronized state compared to the profile during the synchronized state (Fig. 6c): the strongest suppression appeared at around 80 ms after stimulus onset in the desynchronized state (~100 ms in the synchronized state) followed by the quicker rebound activation. On the other hand, brief suppression after onset responses was not apparent in MGB. To quantify these observations, mean spike time in an 80–200 ms time window was compared between states (Fig. 6g–i). Mean spike time in AC was significantly shorter in the desynchronized state compared to that in the synchronized state (*p* < 0.0005, two-tailed *t*-test) (Fig. 6g) whereas there was no significant difference in MGB (*p* = 0.60) (Fig. 6i). Within AC cell classes, although effects of cortical states across cell classes were not statistically significant (F_4,237_ = 0.43, *p* > 0.05), BS3 cells showed a tendency of the reduction in mean spike time (Fig. 6h and Supplementary Table 6). Thus, state-dependent rapid response was observed in AC, not in MGB.

### Cortical desynchronization improves rapid auditory processing

**Figure 7:**
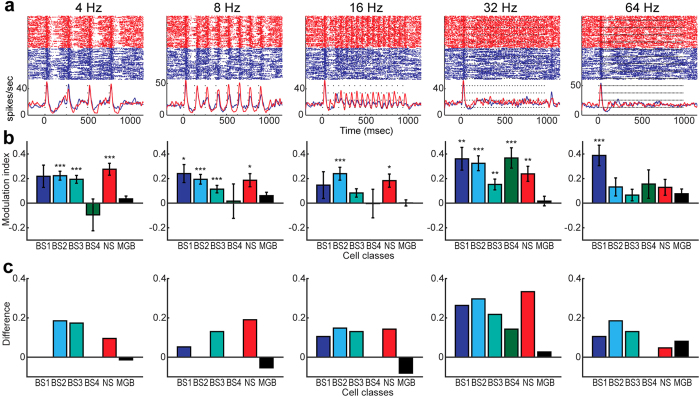
State-dependent temporal processing in AC and MGB. (**a**) Examples of cortical multiunit responses to click train stimuli across different frequencies in the desynchronized (*red*) and synchronized states (*blue*). (**b**) Mean modulation index of vector strength across cell classes. Errors indicate SEM. *p < 0.05, **p < 0.01, ***p < 0.005 (two-tailed *t*-test with Bonferroni correction). (**c**) Difference in a fraction of modulated cells across cell classes. Positive values mean that more cells showed significant modulation (Rayleigh test, *p* < 0.01) during the desynchronized state.

What is the functional consequence of such state-dependent auditory responses ? Based on the finding in Fig. 6, we predicted that the desynchronized state improves temporal processing in AC, but not in MGB. To test this prediction, we analyzed state-dependent responses to temporally structured click trains (Fig. 7). Figure 7a shows population activity (multiunit activity) in cortical infragranular layers, with more precisely resolved click train responses during the desynchronized state. To quantify this trend at the single-unit level, we assessed the vector strength and statistical significance of rate modulation (Rayleigh test, *p* < 0.01) in click train responses across neurons in AC (n = 143) and MGB (n = 68). Improvement of click train responses during the desynchronized state was observed in AC with respect to both a modulation index (Fig. 7b) and a difference in the number of significantly modulated cells between states (Fig. 7c). While the effect varied depending on cell classes and click train frequencies, consistent improvement was observed in AC, but not in MGB. More BS3 and NS cells consistently improved temporal processing across click train frequencies (Fig. 7c) whereas within cortical cell classes, BS4 cells appeared to be sensitive to a particular click train frequency (i.e., 32 Hz) (*p* < 0.005, two-tailed *t*-test with Bonferroni correction) (Fig. 7b). On the other hand, none of tested stimuli significantly improve MGB cells’ responses (*p* = 0.82 at 4 Hz; *p* = 0.19 at 8 Hz; *p* = 1 at 16 Hz; *p* = 1 at 32 Hz; *p* = 0.33 at 64 Hz, two-tailed *t*-test with Bonferroni correction) (Fig. 7b). Thus, the desynchronized state improved temporal processing across AC layers to varying degrees, but not in MGB.

## Discussion

Combining *in vivo* large-scale extracellular recording in the auditory thalamocortical circuit of urethane-anesthetized rats with BF electrical stimulations, we made the following observations: (1) Cortical states are associated with distinct cortical oscillations and spontaneous firing across cortical layers; (2) At onset responses to a single click, neural firing in both AC and MGB became more reliable during the desynchronized state; (3) NS cells show increased onset responses whereas BS cells show reduction during the desynchronized state; (4) Although the reduced response variability was also found subcortically, AC population showed a distinct dynamics of evoked responses, with quicker rebound activity especially in infragranular layers; (5) This rapid processing in the desynchronized state was associated with the improvement of temporal tuning across AC layers, but not in MGB. To the best of our knowledge, this study is the first to characterize state dependence of population activity in both AC and MGB simultaneously, with an emphasis on firing across cortical layers and the temporal structure of evoked population responses. These results are also consistent with the hypothesis that AC plays an important role in integration of external signals with internally generated activity.

Laminar-specific oscillations (Fig. 3) have been observed in several models: *in vitro* neurophysiological experiments in AC identified strong gamma oscillations in superficial layers, with distinct mechanisms between L2/3 and L4^41^,^42^, and auditory evoked gamma oscillations have been also seen *in vivo*^43^. However, the present results are novel with respect to state dependence of laminar-specific gamma oscillations *in vivo*. The reduction in power across frequencies and layers supports the notion that a “desynchronized” state is indeed asynchronous. One notable exception is the increased gamma power in superficial layers (Fig. 3c). Intriguingly, the macaque visual cortex also shows stronger gamma oscillations in superficial layers^44^,^45^ and they are modulated by attention^46^, suggesting a general property across modalities and species. The mechanisms of laminar-specific gamma oscillations are less clear^41^. It would be interesting to investigate how thalamic neurons drive superficial parvalbumin-positive (PV + ) neurons to modulate superficial gamma oscillations. PV + neurons in BF may also play a role in cortical gamma oscillations^47^.

The exact mechanisms underlying the observations reported here are uncertain because of the nature of electrical stimulation. The BF consists of diverse cell classes, including cholinergic, glutamatergic and GABAergic neurons^48^, and so direct effects of electrical BF stimulation on recorded cell populations are likely to be complex. Moreover, other subcortical structures were also likely to be activated antidromically. Despite this, laminar-specific effects of BF stimulations on spontaneous firing in AC are generally consistent with a previous study^25^, where firing rate in superficial pyramidal cells decreases during different types of desynchronization, including electrical stimulation of another cholinergic nucleus (i.e., pedunculopontine tegmental nucleus) and the desynchronized state in an unanesthetized condition. Thus, effects of electrical BF stimulation on cortical population activity in AC may be generalizable. Particularly together with a recent finding in head-restrained behaving mice^24^, the desynchronized states are generally associated with sparser superficial activity and diverse effects in infragranular layers. This picture is also consistent with state-dependent firing in the somatosensory barrel cortex of unanesthetized rodents^7^,^23^ and the primary visual cortex of anesthetized rats^8^.

However, we also noticed several discrepancies. Although we previously observed the reduction in NS cell firing^25^, electrical BF stimulation in the present study did not change spontaneous firing rate of NS cells significantly, implying different mechanisms of cortical desynchronization. For example, assuming that NS cells are PV + neurons, the present BF electrical stimulation may not induce a cortical desynchronization via cholinergic activation of somatostatin-positive neurons^49^. In addition, the desynchronized state seems to be different from that in the mouse visual cortex during locomotion^13^,^26^. Although these discrepancies still remain to be explained, cortical states are further defined by subtle differences in population activity^2^,^50^. Optogenetic approaches will disentangle this complexity of cortical desynchronization mechanisms in future.

While a previous study reported enhanced thalamocortical synaptic transmission in AC of anesthetized rats using nucleus basalis stimulation^39^, the present study is novel by assessing auditory-evoked responses in both AC and MGB and by showing cell type specificity. The reduction in onset evoked responses in AC (Fig. 5) is consistent with observations in behaving rats^11^ and mice^51^,^52^ and the desynchronized somatosensory cortex^6^ although opposite effects were observed in the visual cortex^13^ and lateral geniculate nucleus^52^. Improved spike count reliability in AC and MGB is also consistent with previous studies in the visual system^8^, somatosensory cortex^53^ and many other cortical areas^54^. The reduced response variability in desynchronized cortex may be explained at least partially by a thalamic contribution^55^. Intriguingly, we also observed the increased onset response of NS cells, consistent with the idea that sparse and reliable cortical activity is realized by the dominant inhibition^56^,^57^. It will be important to examine how thalamic cells interact with cortical cells in a cell type-specific manner.

What is special about AC compared to MGB in terms of state-dependent processing ? The quicker rebound activity in the desynchronized state emerged cortically. Intriguingly, attentional modulation of firing rate is typically observed in the late component rather than onset responses^18^, and depends on the prefrontal cortical activity^58^. The stronger tendency of the rebound activity in infragranular layers, especially BS3 cells, (Fig. 6h) supports a recently proposed model in the somatosensory cortex^59^. In addition to long range cortical feedback, the increased evoked responses of NS cells during the desynchronized state (Fig. 5c) may also contribute to this quick rebound firing^56^. Thus, interactions between local and distal components in cortical circuits are likely key for a mechanistic understanding of state-dependent modulation at the late phase.

Although the functional role of this quicker rebound activity in auditory processing is uncertain, one functional implication is the improvement of temporal tuning (Fig. 7). While laminar-dependent spectral^27^,^28^ and temporal tuning^29^ were previously investigated in AC, the current study offers a novel insight into the functional role of global brain states in auditory cortical processing. In this regard it will be important to investigate how cortical states affect rapid auditory processing in behaving animals and how abnormality in temporal tuning is associated with dyslexia^35^ and age-related slowness of auditory processing^36^ at the level of neural circuits. Because BF has long been implicated in attention, arousal, learning and memory^37^,^38^,^60^, BF stimulation may be beneficial to restore rapid auditory processing.

## Methods

### Animals

Five adult Sprague-Dawley rats (male, 267–357 g) were used. Experiments were performed in accordance with the UK Animals (Scientific Procedures) Act of 1986 Home Office regulations and approved by the Home Office (PPL 60/4217).

### Surgical procedures

Animals were anesthetized with 1.5 g/kg urethane. Lidocaine (2%, 0.1–0.3 mg) was also administered subcutaneously at the site of incision. After attaching a head-post in the frontal region with bone screws, one of which was used as an electrode for cortical electroencephalograms (EEGs), the animal was placed in a custom head restraint that left the ears free and clear. Two additional bone screws were implanted in the cerebellum as ground. Body temperature was maintained at 37 °C with a feedback temperature controller (40–90–8C, FHC). All electrophysiological experiments described below were performed in a single-walled soundproof box (MAC-3, IAC Acoustics) with the interior covered by 3 inches of acoustic absorption foam.

### BF stimulation

The bone above the basal forebrain (BF) was removed, and a concentric bipolar stimulation electrode (20–50 kΩ at 1 kHz, SNE–100; David Kopf Instruments) was implanted into the right BF (0.8 mm posterior from the bregma, 2.5 mm lateral from the midline, 7.0 – 8.7 mm from the dorsal surface of the neocortex). Optimal stimulation depth was identified by inducing an desynchronized state of cortical EEGs with a 1 sec pulse train (100 Hz, 200 μs duration, 50 μA) (Model 2100, A–M Systems)^8^.

### Electrophysiological recording

After reflecting the left temporalis muscle, the bone above the left medial geniculate body (MGB) (5.8 mm posterior from the bregma, 3–4 mm lateral from the midline) and over the left auditory cortex (5–6 mm posterior from the bregma, around the temporal junction between the parietal and squamosal bones)^61^ was removed and a small duratomy for each site was carefully performed. Two 32 channel silicon probes (A1 × 32–10 mm–50–177–A32, NeuroNexus Technologies) were inserted slowly (2 μm/sec or slower) with a motorized manipulator (DMA-1511, Narishige) or a manual micromanipulator (SM-25A, Narishige), with one probe to the ventral part of MGB (MGv, 6.0 mm from the dorsal surface of the cortex) and the other to the auditory cortex (AC) (1700 – 1770 μm from the surface). While the primary auditory cortex was targeted, because tonotopic maps were not systematically mapped, in the present study we deliberately referred the recorded cortical area as AC. During recording, the brain was covered with 1% agar/0.1 M phosphate buffered saline to reduce pulsation and to keep the cortical surface moisture. Broadband signals (0.07 Hz – 8 kHz) from the silicon probes were amplified (1000 times) (Plexon, HST/32V-G20 and PBX3), digitized at 20 kHz and stored for offline analysis (PXI, National Instruments).

A typical recording schedule was as follows: after insertion of the probes and an additional waiting period (up to 30 min), recording was started with a silent period (at least 5 min), followed by sound presentations with and without BF stimulations (see below) and ended with another silent period (at least 5 min). During the latter silent period, BF stimulation (1 sec) was applied manually to induce the desynchronized state. By confirming recovery of cortical states to the synchronized state, then another stimulation was applied. This period was used to assess state-dependent spontaneous activity (Figs 3 and 4).

### Acoustic stimulation with BF stimulation

Acoustic stimuli were generated digitally (sampling rate 97.7 kHz, TDT3, Tucker-Davis Technologies) and delivered in free-field through a calibrated electrostatic loudspeaker (ES1) located ~10 cm in front of the animal. Calibration was conducted using a pressure microphone (PS9200KIT-1/4, ACO Pacific Inc) close to the animal’s right ear. Acoustic stimuli consisted of brief clicks (5 ms long broadband noise with 1 ms cosine ramps, 10 dB steps, 0–80 dB SPL) and 1 sec long repetitive click trains (5 ms broad band noise with 1 ms cosine ramps, 4–64 Hz at 70 dB SPL). To assess state-dependent auditory processing, we adopted a block design^8^ (Fig. 1c): in “no stimulation” blocks, only auditory stimuli were presented whereas in “stimulation” blocks, 1 sec BF stimulation was applied at the beginning of every trial, followed by sound presentations. After the stimulation block, no BF and auditory stimuli were applied for 30 sec to confirm recovery of cortical states to the synchronized state. Although other acoustic stimuli (short tone pips) were also presented, these were not analyzed as they were not combined with BF stimulation. The present analysis focused on evoked responses to clicks at 70 dB SPL for direct comparisons to the click train stimulation at 70 dB SPL.

### Histology

After electrophysiological experiments, rats were perfused transcardially with physiological saline followed by 4% paraformaldehyde/0.1 M phosphate buffer, pH 7.4. After an overnight postfixation in the same fixative, brains were cut into 100 μm coronal sections with a sliding microtome (SM2010R, Leica), and the sections were collected and placed in 0.1 M phosphate buffered saline (PBS). For verification of silicon probe and stimulation electrode tracks, the free-floating sections were counterstained with NeuroTrace (1/500, N-21480, Life Technologies) in PBS with 0.1% Triton X-100 for 20 min at room temperature. The sections were mounted on gelatin-coated slides and cover-slipped with antifade solutions.

### Signal processing

All spike detection and sorting took place off-line using freely available software (EToS version 3, http://etos.sourceforge.net; Klusters, http://klusters.sourceforge.net)^62^,^63^. To avoid clustering in high dimensional space, only subsets of channels were processed depending on recording quality. For thalamic recordings, only channels which clearly showed auditory evoked responses were processed. After automatic and manual clustering, unit isolation quality was assessed by measuring “isolation distance”^64^; only cells with value ≥20 were further analyzed. Spike train analysis and other signal processing were performed with MATLAB (Mathworks). To analyze local field potentials (LFPs), low pass filtered (<200 Hz) signals were resampled at 1 kHz. The power spectral density was computed with Chronux (http://chronux.org).

### Depth evaluation

We aligned the depth of recorded positions across experiments as follows (Fig. 2). Current source density (CSD) analysis was employed to identify thalamic recipient layers. CSD profiles were generated from depth profiles of average LFPs using previously described methods^27^,^65^. First, we duplicated LFPs corresponding to the uppermost and lowermost channels. Second, LFPs were smoothed across spatially adjacent channels to reduce high spatial-frequency noise components:


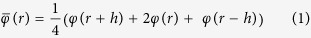


where *φ*(*r*) is the LFP at depth *r*, and *h* is the sampling interval (50 μm in this case). Next, we calculated the second derivative:


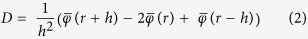


Then the channel that showed the largest sink signal was recognized as a thalamic recipient layer, presumably L3/4, and relative depths were determined accordingly (Fig. 2a).

We further estimated the depth of spike-sorted units^25^,^27^. Somatic location was estimated as the recording site with maximum trough-to-peak amplitude based on mean spike waveforms (Fig. 2b).

### Single-unit classification

Cortical spike-sorted units were classified based on trough-to-peak time of average spike waveforms (Fig. 2c). 0.55 ms was chosen to discriminate between broad spiking (BS) and narrow spiking (NS) cells. The latter cell class may correspond to parvalbumin-positive fast spiking neurons^66^. BS cells were further classified into four groups based on the estimated somatic location (see above): 0 ~ 200 μm (BS1), −300 ~ 0 μm (BS2), −700 ~ −300 μm (BS3), and −1100 ~ −700 μm (BS4) (Fig. 2c). These subgroups may correspond to different cortical layers (L2/3, L3/4, L5 and L6)^40^.

### UP state detection

UP states were detected in Fig. 4e and detailed procedures were described elsewhere^27^. Briefly UP states were detected from the smoothed cortical multi-unit activity (MUA) (summed population activity across channels in AC, smoothed with a 10 ms Gaussian kernel), during periods without stimulus presentation. UP state onsets were determined if the following two conditions were fulfilled: 1) the smoothed MUA crossed above a threshold defined as the geometric mean of MUA over all spontaneous activity. 2) Mean MUA rate was below 20% of the threshold value for at least 100 ms before onset. We further analyzed only UP states for which mean MUA remained above the threshold over a 200 ms window after onset. Spontaneous firing during UP states was also estimated in this 200 ms window.

### Spike train statistics

For comparison of firing properties (e.g., mean spike count *μ*, variance σ^2^, Fano factor σ^2^/ *μ*, and vector strength) between the desynchronized and synchronized states, we computed the modulation index as follows: (DESYNC – SYNC)/(DESYNC + SYNC), where DESYNC and SYNC are firing properties in the desynchronized and synchronized states, respectively. For comparisons of spontaneous activity, 3 sec time windows before and after BF stimulation were used.

To quantify click train responses, circular statistics were applied^67^. Spikes until 50 ms from the first click onset were not included for this analysis to avoid spuriously high vector strength due to initial onset responses. To quantify to what extent spike timing aligned to individual clicks of the click train stimulus, a vector strength (the length of the mean resultant vector) was computed using the following formulae^68^:


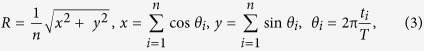


where *n* is the total number of spikes, *t*_*i*_ is the time of spike occurrence from a click onset, and *T* is the inter-click interval. Statistical significance of phase-locked responses to clicks was assessed by the Rayleigh test and a p-value of less than 0.01 was considered significant.

### Correlation analysis

To compute spike count correlation of spontaneous activity before and after BF stimulation (Fig. 4), spike trains from two simultaneously recorded single units were extracted 3 sec before and after 1-sec BF stimulations and two vectors were constructed with 1 ms bin and 5 ms Gaussian kernel. Then the Pearson correlation coefficient was computed.

### Statistical analysis

Data were presented as mean ± SEM. For multiple comparisons, two-way ANOVA was performed, followed by post-hoc Tukey’s honest significant difference (HSD) test. In Fig. 4, Kruskal-Wallis test and signed rank test were applied. In Figs 5 and 7b, two-tailed *t*-tests with Bonferroni correction were performed to compare to zero. All statistical analyses were conducted using MATLAB.

## Additional Information

**How to cite this article**: Sakata, S. State-dependent and cell type-specific temporal processing in auditory thalamocortical circuit. *Sci. Rep.* **6**, 18873; doi: 10.1038/srep18873 (2016).

## Supplementary Material

Supplementary Information
