# Supplementary material for: State-dependent and cell type-specific temporal processing in auditory thalamocortical circuit
Source: Sci Rep. 2016 Jan 5;6:18873. doi: 10.1038/srep18873 (PMC4700423; doi:10.1038/srep18873)
Supplement: Supplementary Information [file srep18873-s1.pdf]

Supplementary Information

State-dependent and cell type-specific temporal processing in auditory thalamocortical circuit

Shuzo Sakata
Strathclyde Institute of Pharmacy and Biomedical Sciences
University of Strathclyde
161 Cathedral Street, Glasgow G4 0RE, UK

Address correspondence to:
Shuzo Sakata
161 Cathedral Street, Glasgow G4 0RE, UK
email: shuzo.sakata@strath.ac.uk
tell: +44-141-548-2156

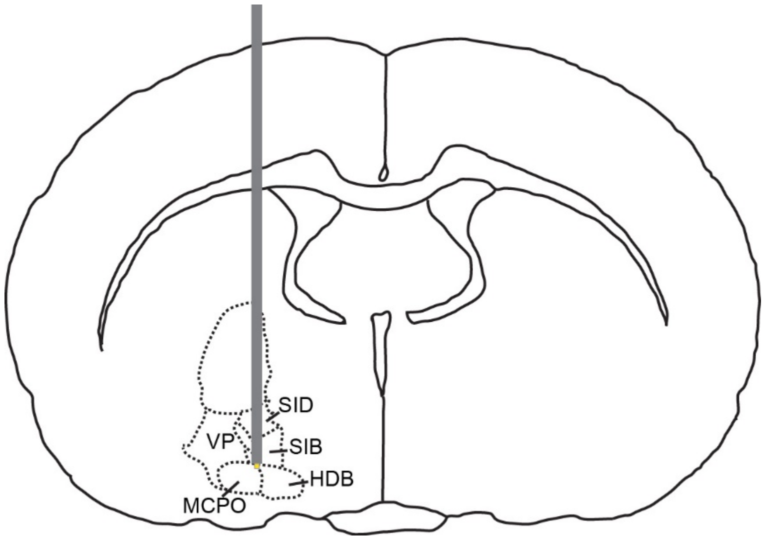

Figure S1 A schematics of a stimulation electrode insertion into the basal forebrain. HDB, nucleus of the horizontal limb of the diagonal band. MCPO, magnocellular preoptic nucleus. SIB, substantia innominate, basal part. SID, substantia innominate, dorsal part. VP, ventral pallidum.

	BS1	BS2	BS3	BS4	NS	MGB
BS1		0.204	0.039	0.901	0.338	0.882
BS2			0.999	0.015	1.000	0.487
BS3				0.001	0.994	0.051
BS4					0.037	0.206
NS						0.715
MGB						

Table S1 *P* values of post-hoc Tukey's honest significant different (HSD) test in **Figure 4b**. Red, $p < 0.05$.

	BS1	BS2	BS3	BS4	NS	MGB
BS1		0.001	0.000	1.000	0.002	0.002
BS2			0.857	0.008	1.000	0.914
BS3				0.000	0.881	0.047
BS4					0.015	0.027
NS						0.952
MGB						

Table S2 *P* values of post-hoc HSD test in **Figure 4c**. Red, $p < 0.05$.

		AC-AC		MGB-MGB		AC-MGB	
		Sync	Desync	Sync	Desync	Sync	Desync
AC-AC	Sync		0.000	0.116	1.000	0.000	0.000
	Desync			0.000	0.000	0.887	0.006
MGB-MGB	Sync				0.415	0.000	0.000
	Desync					0.000	0.000
AC-MGB	Sync						0.254
	Desync						

Table S3 *P* values of post-hoc HSD test in **Figures 4f**. Red, $p < 0.05$.

		BS-BS		BS-NS		NS-NS	
		Sync	Desync	Sync	Desync	Sync	Desync
BS-BS	Sync		0.000	0.000	0.879	0.000	0.000
	Desync			0.000	0.217	0.000	0.016
BS-NS	Sync				0.000	0.003	0.000
	Desync					0.031	0.922
NS-NS	Sync						0.000
	Desync						

Table S4 *P* values of post-hoc HSD test in **Figures 4g**. Red, $p < 0.05$.

		BS-MGB		NS-MGB	
		Sync	Desync	Sync	Desync
BS-MGB	Sync		0.000	0.393	0.001
	Desync			0.000	0.000
NS-MGB	Sync				0.326
	Desync				

Table S5 *P* values of post-hoc HSD test in **Figures 4h**. Red, $p < 0.05$.

		BS1		BS2		BS3		BS4		NS	
		Sync	Desync	Sync	Desync	Sync	Desync	Sync	Desync	Sync	Desync
BS1	Sync		1.000	0.977	0.800	0.912	0.034	0.096	0.001	0.332	0.008
	Desync			1.000	0.994	1.000	0.389	0.289	0.012	0.832	0.126
BS2	Sync				1.000	1.000	0.198	0.335	0.009	0.877	0.054
	Desync					1.000	0.692	0.567	0.031	0.996	0.254
BS3	Sync						0.036	0.322	0.006	0.835	0.017
	Desync							0.958	0.244	1.000	0.967
BS4	Sync								0.999	0.891	1.000
	Desync									0.176	0.777
NS	Sync										0.885
	Desync										

Table S6 *P* values of post-hoc HSD test in **Figures 6h**. Red, $p < 0.05$.
